# Impact of Wearable Technology on Heart Failure Management

**DOI:** 10.31661/gmj.v13i.3469

**Published:** 2024-09-11

**Authors:** Negar Jafari, Sahar Yousefi Ghalati, Venus Shahabi Raberi, Saba Moalemi, Arash Amin

**Affiliations:** ^1^ Department of Cardiology, School of Medicine, Urmia University of Medical Sciences, Urmia, Iran; ^2^ School of Nursing and Midwifery, Shiraz University of Medical Sciences, Shiraz, Iran; ^3^ International Training Fellow of cardiology, Wwl Nhs Trust, UK; ^4^ Department of Managment, Shiraz Branch, Islamic Azad University, Shiraz, Iran; ^5^ Lorestan Heart Center (Madani Hospital), Lorestan University of Medical Sciences, Khorram-Abad, Lorestan, Iran

**Keywords:** Heart Failure, Myocardial Infarction, Wearable Technology, Biosensor, Monitoring, Smartwatch, Artificial Intelligence

## Abstract

Background: Heart failure (HF) is a chronic and progressive condition that its management presents significant challenges in both clinical settings and patient self-care. Recent advances in wearable technology offer promising solutions to these challenges by enabling continuous monitoring, early detection of clinical deterioration, and personalized care. This review aims to critically evaluate the impact of wearable technology on HF management Materials and Methods: This narrative systematic review was conducted across multiple databases, including PubMed, Web of Science, and the Cochrane Library, to identify relevant studies published between 2010 and 2024. Studies on wearable devices for HF management and monitoring were included if they reported on clinical trials and provided data on integration into clinical workflows. Studies on other conditions or without original research data or Non-English papers were excluded. Results: Nine studies were evaluated in this study that were focusing on a variety of technologies ranging from consumer-grade fitness trackers to specialized bioimpedance sensors and wearable cardioverter-defibrillators. These studies demonstrate the potential of wearables to continuously monitor important health metrics, which can lead to early intervention and personalized care. However, there are still challenges to be addressed, including concerns about data accuracy, patient adherence, small sample sizes, and the incorporation of wearable data into clinical practice. While consumer devices are more accessible, their accuracy in a clinical setting is uncertain, while more advanced devices like the “Volum” monitor and BioZ sensors show promise but require further validation. Conclusion: This review highlights the growing importance of wearable technologies in HF management, actionable insights that can prevent disease progression. However, significant challenges remain, including the need for further validation, device optimization, and data standardization before routine clinical practice. Future advancements should focus on improving device accuracy, patient adherence, and data security, while ensuring equitable access to these technologies.

## Introduction

Wearable technology has become an increasingly important tool in the management of chronic health conditions, including heart failure (HF), which remains a leading cause of morbidity and mortality worldwide as a major complication of myocardial infarction (MI) and other cardiac diseases [1][2][3]. HF is a complex clinical syndrome that results from structural or functional impairment of ventricular filling or ejection of blood, leading to insufficient blood supply to meet the body’s demands [3].

The traditional approach to managing HF often relies on patient self-monitoring and periodic clinical assessments, which can be challenging to optimize and often fail to detect early signs of decompensation [4][5].

The integration of wearable technology into HF management represents a significant advancement in patient care, offering continuous, real-time monitoring of physiological parameters, early detection of disease exacerbation, and enhanced patient engagement. Also, it can be useful in tertiary prevention of MI [6].

Smartwatches are the most popular wearable devices; however, patches and other sensor-equipped garments can monitor a variety of cardiovascular parameters [7][8]. Due to advancements in technology, traditional implants such as defibrillators have transformed into wearable devices [9][10]. These devices are particularly beneficial in HF management as they can detect subtle changes in physiological status that might indicate an impending HF exacerbation, allowing for timely intervention and potentially preventing hospitalizations [11]. Additionally, the use of artificial intelligence (AI) and machine learning algorithms to analyze data from these devices has the potential to further improve the accuracy and predictive power of HF management strategies [12].

The objectives of this review are providing a comprehensive overview of the current state of wearable technology in HF management and evaluate the evidence supporting the use of these technologies in clinical practice, addressing both their potential benefits and the challenges that remain.

## Materials and Methods

Design

A systematic review utilizing narrative methods was conducted to examine the existing evidence. Due to a review methodology [13] was implemented to elucidate the types of wearable devices and their current application in HF management.

Search Strategy

A comprehensive literature search was conducted to identify relevant studies on the impact of wearable technology on HFmanagement. The following electronic databases were used for the search: PubMed, Web of Science, and the Cochrane Library. The search was conducted using a combination of keywords and MeSH terms related to wearable technology and HF management. Specific keywords included " wearable technology or smart watch or wearable sensor or wearable electronics or wearable computers or wearable device and "heart failure". Boolean operators such as "AND" and "OR" were utilized to refine the search results. The search was limited to peer-reviewed articles published in English from January 2010 to July 2024, ensuring that the review included the most recent and relevant studies.

Inclusion/Exclusion Criteria

Studies were selected for inclusion based on the following criteria:

Inclusion Criteria:

1. Studies that focused on the use of wearable devices specifically designed for the management or monitoring of HF.

2. Articles reporting on clinical trials that including randomized clinical trials and quasi-experimental studies evaluated the effectiveness of wearable technology in improving clinical outcomes, patient adherence, or quality of life in HF patients.

3. Studies that provided data on the integration of wearable technology into clinical workflows and its impact on healthcare delivery.

4. Studies described in full-text papers.

Exclusion Criteria:

1. Studies that focused on wearable technology for conditions other than HF, unless the technology was specifically used in a HF subgroup.

2. Articles that did not include original research data, such as editorials, commentaries, or opinion pieces.

3. Studies with incomplete data or those not published in peer-reviewed journals.

4. Non-English language studies were excluded to maintain consistency in the analysis.

Data Extraction

Data from the selected studies were extracted systematically using a standardized data extraction form. The following information was collected from each study:

Study characteristics, including author(s), year of publication, study design, and sample size.

Details of the wearable technology used, including the type of device, parameters monitored, and duration of use.

Key outcomes related to HF management, such as hospital readmission rates, mortality, patient adherence to treatment, and quality of life measures.

Data were then analyzed and synthesized to identify trends, strengths, and limitations across the studies. A qualitative synthesis was conducted to integrate the findings and provide a comprehensive overview of the impact of wearable technology on HF management.

Study Selection

Initially, two authors independently evaluated all titles and abstracts identified as relevant to the systematic review. Subsequently, these abstracts were further assessed for eligibility by the same two authors. then, the full texts of studies that met the eligibility criteria were obtained and reviewed by another author, based on predefined inclusion and exclusion criteria. Any disagreements were resolved through discussion until consensus was reached. To ensure a thorough search, the references of recent related reviews and primary studies were also manually screened for additional relevant studies.

## Results

**Figure-1:**
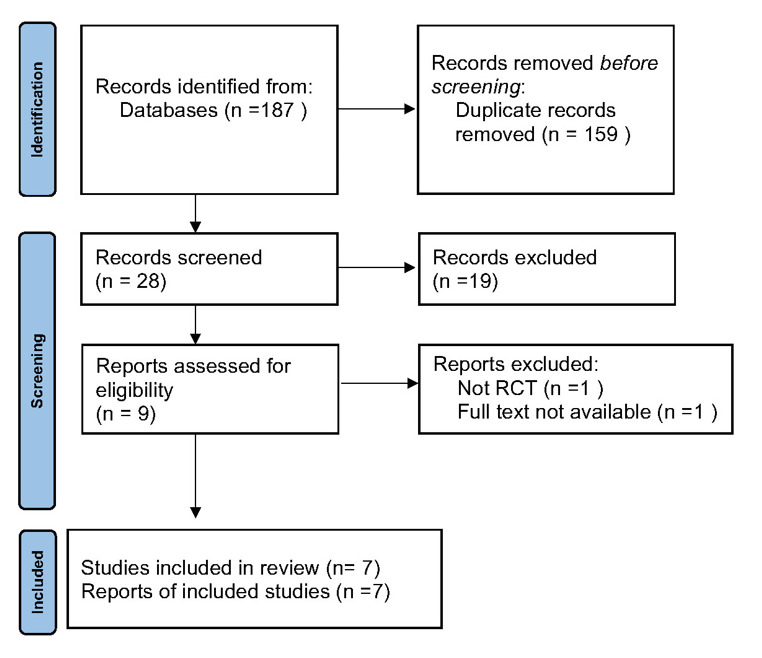
Figure 1. The PRISMA flow diagram illustrating the screening process of studies.

**Table:** Table1. Summary of the Type of Wearable Device, Study Population, Key Findings, and Limitations

Study	Type of Wearable Device	Study Population	Key Findings	Limitations
Seysha Mehta, 2020 [15]	Microsoft Band fitness tracker	23 HF patients, 6 cardiologists	Wearable data influenced cardiologists' perceptions of health status.	Small sample size; technical issues resulted in incomplete data; data reviewed post-visit rather than in real-time.
Seulki Lee, 2015 [16]	Wearable Bio-impedance (BioZ) sensor	8 HF patients	BioZ sensor useful for fluid balance measures for in-hospital monitoring.	Limited to hospital settings; small sample size; potential confounding factors.
Santiago F. Scagliusi, 2023 [17]	"Volum" wearable bioimpedance device	1 HF patient, 1 healthy control	Suggested a new device for noninvasive monitoring of bioimpedance evolutioni n the HF patient.	Very small study population; inconsistent bioimpedance readings possibly due to improper placement or other uncontrolled variables.
Jeffrey E. Olgin, 2020 [18]	Wearable Cardioverter-Defibrillator (WCD)	1767 HF patients (989 WCD and 778 control group)	WCD is preventing sudden cardiac death in HF patients.	Details on the study population and specific results are limited; observational nature of the study; patient compliance issues.
Isabell Anna Just, 2022 [19]	Myosuit (focus on movement therapy)	20 HF patients ( 10 Myosuit and 10 control group)	Myosuit is safe and HF patients use it in in rehabilitation programs.	Lack of details on wearable devices used, if any; general limitations related to therapy-based studies.
Jessica R. Golbus, 2023 [20]	Fitbit device	425 HF patients	increases in step count tracked by a wearable device over time could hold clinical importance.	Variability in data quality and interpretation due to diverse devices used.
Raj M. Khandwalla, 2019 [21]	Philips Actiwatch Spectrum (pedometers/ accelerometers)	136 HF patients	Wearable accelerometers will be utilized to understand how the medication influences physical activity and sleep patterns.	Need for extensive training of both participants and clinical research teams, short duration may not capture the long-term effects of treatment on myocardial function and sleep

Figure-1. shows the PRISMA 2020 flow diagram [14] of this study. Overall, 187 papers were found in databases however 159 of them were duplicated and removed. Following the review of titles and abstracts, the search identified 28 documents as potentially relevant. Of these, 9 papers met the inclusion criteria after a thorough full-text review so 2 papers were excluded. The remaining 7 studies on wearable technology in HF management are summarized in Table-1.

1. Overview of Wearable Technology in HF Management

Wearable technology has emerged as a transformative tool in the management of HF, offering continuous monitoring and real-time data collection that can significantly improve patient outcomes [22].

1.1. Types of Wearable Devices

Various types of wearable devices are utilized in HF management, each offering distinct functionalities tailored to different aspects of the disease [3]. Smartwatches are among the most commonly used devices, capable of tracking heart rate, physical activity, and sleep patterns. These devices are widely accessible and user-friendly, making them popular for daily health monitoring [7]. For example, smartwatches like the Apple Watch and Fitbit have integrated electrocardiogram (ECG) capabilities, allowing users to monitor their heart rhythms and potentially detect arrhythmias [8][23].

Mehta, et al. [15] showed Consumer-grade fitness trackers like the Microsoft Band, used to provide continuous, objective data on patient activity levels and general health metrics.

Biosensors represent another critical category of wearable technology in HF management. These sensors are often embedded in patches or wristbands and are designed to monitor a variety of physiological signals, including bioimpedance, blood oxygen levels, and respiratory rate [11][24].

Lee, et al. [16] demostarated Wearable Bio-impedance (BioZ) sensors, designed to monitor fluid accumulation in congestive HF (CHF) patients, offering a more specialized measurement tool compared to general fitness trackers. Also, Scagliusi, et al. [17] presented the "Volum" wearable bioimpedance devices, which are portable and designed for continuous, remote monitoring of bioimpedance, specifically targeting fluid retention in CHF patients.

Moreover, Khandwalla, et al. [21] reported activity sensors such as pedometers or accelerometers, used to evaluate the impact of medications on the physical activity and sleep pattern in HF patients. Wearable devices have proven useful in tertiary prevention such as cardioverter-defibrillators and physical therapy [10][24].

Olgin, et al. [18] demonstrated The ZOLL Life® Wearable Cardioverter-Defibrillator (WCD) is used for preventing sudden cardiac death by detecting and correcting life-threatening arrhythmias in real-time. On the other hand, Just, et al.[19] reported Myosuit is safe and useful for movement therapy in managing advanced HF symptoms. The Myosuit, a soft and wearable exoskeleton-type robot created by MyoSwiss AG, is safe and useful for movement therapy in managing advanced HF symptoms [25]. The data collected from these devices are often transmitted to healthcare providers, enabling remote monitoring and timely interventions [12].

1.2. Technological Advancements

Recent years have seen significant advancements in wearable technology, particularly in the context of HF management [26]. One of the most notable innovations is the development of bioimpedance monitoring devices. These devices measure the impedance of body tissues to detect changes in fluid levels, which is particularly important in monitoring HF patients who are prone to fluid retention [24]. The BioZ sensors and the "Volum" device have demonstrated high accuracy in detecting fluid accumulation in HF patients, providing continuous and non-invasive real-time monitoring enabling more accurate and timely clinical interventions [16][17].

Another significant advancement is the integration of AI and machine learning algorithms into wearable devices [12]. These technologies enable the analysis of large datasets generated by wearables, facilitating more accurate predictions of HF exacerbations and personalized treatment plans [22].

The miniaturization and improved connectivity of wearable devices have also enhanced their utility in HF management. Modern devices are smaller, more comfortable, and equipped with wireless connectivity, allowing for seamless data transmission to healthcare providers [11]. This has enabled the development of telemedicine platforms that integrate wearable device data into electronic health records, providing a comprehensive view of the patient's health and facilitating remote care [26].

Furthermore, WCDs represent a significant advancement in the prevention of sudden cardiac death by offering real-time responses to life-threatening arrhythmias [6][10][18] Additionally, the use of robotic exoskeleton-assisted mobilization for patients with advanced HF has proven to be safe, feasible, and patients well-tolerated [19][27].

2. Challenges and Limitations

Despite the many benefits of wearable technology in HF management, several challenges and limitations hinder its widespread adoption and effectiveness [7]. These challenges span technical, ethical, and economic dimensions, each of which must be addressed to fully realize the potential of these technologies in clinical practice [3].

2.1. Technical Challenges

One of the primary technical challenges associated with wearable technology is accuracy. Many wearable devices rely on sensors that can be prone to errors due to various factors, such as sensor misalignment, motion artifacts, or environmental conditions [23]. For instance, bioimpedance sensors used to monitor fluid retention in HF patients can be affected by changes in body posture or movement, leading to inaccurate readings [24]. Similarly, heart rate monitors embedded in smartwatches may provide less accurate data during periods of high physical activity or when the device is not worn correctly [7]. The accuracy of these devices is crucial for clinical decision-making, and any errors can lead to incorrect diagnoses or inappropriate treatments [3][28].

Data security is another significant concern with wearable devices. These technologies generate and transmit vast amounts of sensitive health data, which are often stored on cloud servers or shared with healthcare providers [29]. This data is vulnerable to breaches and unauthorized access, raising concerns about patient privacy and the potential misuse of personal health information [30][31]. Ensuring robust encryption and secure data transmission protocols is essential to protect patient data from cyber threats [32][33]. Battery life is also a critical limitation of current wearable technologies [34]. Many wearable devices require frequent recharging, which can be inconvenient for users, especially those with chronic conditions like HF who rely on continuous monitoring [35].

For example, bioimpedance monitoring devices and smartwatches that track heart function often need to be recharged every few days, which may lead to interruptions in data collection and gaps in monitoring [21][26]. Improving battery efficiency and developing low-power sensors are ongoing challenges in the design of wearable technology [3][36].

2.2. Cost and Accessibility

Cost-effectiveness is a significant barrier to the widespread adoption of wearable technology in healthcare. While wearables can provide valuable health insights, the cost of these devices can be prohibitive for many patients, particularly those from low-income backgrounds or those without adequate health insurance [37][38]. Even less expensive devices, such as smartwatches and fitness trackers, may still be out of reach for some individuals, especially when considering the additional costs of maintenance, such as software updates and battery replacements [39]. Accessibility is another critical issue, particularly in rural or underserved areas where access to healthcare technology is limited [11][40]. Patients in these areas may not have the necessary infrastructure, such as reliable internet connectivity, to support the use of wearable devices that require continuous data transmission [41]. Additionally, there may be a lack of digital literacy among some patient populations, making it difficult for them to effectively use and benefit from wearable technology [40][42].

2.3. Ethical and Privacy Concerns

The widespread use of wearable technology raises several ethical and privacy concerns. One of the most pressing issues is the consent and autonomy of patients [43]. While wearables can empower patients by providing them with detailed insights into their health, there is also the risk of patients feeling pressured to use these devices by healthcare providers or insurers [44].

In some cases, the data generated by wearables could be used to influence insurance premiums or access to healthcare, potentially leading to discrimination against individuals based on their health data [45][46]. Data privacy is another major concern. Wearable devices collect and transmit large volumes of personal health information, which could be exposed to third parties without the patient's explicit consent [30][47]. This raises questions about who owns the data and how it can be used. In many jurisdictions, the legal framework governing the use of health data collected by wearables is still underdeveloped, creating uncertainties about data protection and the rights of patients [48]. Ensuring that patients are fully informed about how their data will be used and stored, and that they have control over this data, is crucial for maintaining trust in these technologies [31]. Furthermore, the potential for surveillance and monitoring by employers, insurers, or even governments is a growing concern. Wearables can track a wide range of activities, including physical movements, sleep patterns, and even location data. If this information is used without adequate oversight, it could lead to invasive monitoring practices that infringe on individual privacy and freedom [46][49].

3. Future Directions and Research Gaps

The future of wearable technology in HF management is promising, with numerous innovations on the horizon, emerging research opportunities, and the potential for significant policy changes [50]. As wearable technologies continue to evolve, their integration into clinical practice will likely become more seamless, offering even greater benefits for patients and healthcare providers alike [7]. Emerging technologies in wearable devices for HF management are expected to further enhance the precision, functionality, and usability of these tools [11].

One such innovation is the development of multi-sensor platforms, which integrate various sensors into a single device. These platforms can simultaneously monitor a range of physiological parameters, such as heart rate, bioimpedance, respiratory rate, and oxygen saturation, providing a comprehensive picture of a patient’s health in real-time [51]. The integration of these data streams with AI and machine learning algorithms will enable more accurate predictions of HF exacerbations and personalized treatment [12]. Another exciting development is the advent of flexible and stretchable electronics, which are being incorporated into wearable devices to improve comfort and usability [52]. These materials allow for the creation of wearables that conform more naturally to the body’s contours, reducing discomfort and increasing patient adherence [53]. These advancements are particularly relevant for long-term monitoring, as they minimize the inconvenience associated with traditional rigid devices [7]. Additionally, implantable biosensors are being developed to provide continuous monitoring from within the body, offering even more accurate data and potentially reducing the need for external devices [54]. Telemedicine integration is another significant trend, as wearable devices increasingly interface with telehealth platforms [22]. This integration enables continuous remote monitoring, allowing healthcare providers to track patient data in real-time and intervene promptly when necessary [55]. The COVID-19 pandemic has accelerated the adoption of telemedicine, and wearables are set to play a crucial role in this ongoing shift towards remote healthcare delivery [22].

Despite the progress in wearable technology for HF management, several research gaps remain that need to be addressed to fully realize the potential of these devices [7]. Another research gap lies in the standardization of data collected by wearable devices. Currently, there is significant variability in how data from different devices are collected, processed, and interpreted, which can lead to inconsistencies in clinical decision-making [56]. Establishing standardized protocols for data collection and analysis is essential to ensure that wearables can be reliably used in clinical practice [57]. Additionally, more research is needed to explore the cost-effectiveness of wearable technologies in HF management. While wearables have the potential to reduce hospital readmissions and improve patient outcomes, the initial costs of these devices and their integration into healthcare systems can be significant [58]. Studies that assess the economic impact of wearables, including cost-benefit analyses, will be crucial in determining their broader adoption in clinical practice [37][59].

## Conclusion

The integration of wearable technology into HF management represents a significant advancement in the monitoring and treatment of this chronic condition [22]. This comprehensive review has explored various aspects of wearable technology, including the types of devices available, their clinical impact, the challenges they face, and the future directions for this rapidly evolving field [3]. Wearable technology in HF management encompasses a wide range of devices, from smartwatches and biosensors to advanced implantable devices [6].

These technologies offer continuous monitoring of critical physiological parameters, such as heart rate, rhythm, and bioimpedance, which are essential for managing HF [7][15].

Smartwatches and biosensors provide accessible, non-invasive monitoring options, while implantable devices offer more sophisticated monitoring capabilities and therapeutic interventions, such as cardiac resynchronization and defibrillation [10][18].

The integration of AI and machine learning algorithms further enhances the predictive capabilities of these devices, enabling timely interventions that can prevent adverse events [12]. Additionally, wearable devices have been associated with improved patient engagement and adherence to treatment plans, contributing to better management of HF and potentially reducing mortality [17]. However, despite these promising benefits, wearable technology faces several challenges that must be addressed to fully integrate these devices into clinical practice [7]. Technical challenges, such as accuracy, data security, and battery life, remain significant obstacles [3][36][47]. The accuracy of wearable sensors can be affected by various factors, leading to potential errors in data interpretation and clinical decision-making [24]. Moreover, concerns regarding data privacy and security are paramount, given the sensitive nature of the health data collected by these devices [29].

Ethical considerations, such as patient consent and the potential for surveillance, also need to be carefully managed to maintain patient trust and autonomy [43]. Economic and accessibility issues further complicate the widespread adoption of wearable technology [37].

The high costs associated with advanced wearable devices and the lack of reimbursement policies are significant barriers, particularly for patients from low-income backgrounds or those in underserved areas. [6][37] The findings from this review underscore the burgeoning role of wearable technologies in HF management, highlighting both their potential and current limitations. The studies by Mehta et al., [15] Lee et al., [16] and Scagliusi et al. [17] all point to the promising advancements in non-invasive, real-time monitoring of HF, yet they also there is the need for further validation and optimization before these technologies can be fully integrated into clinical practice. The continuous data provided by these devices could significantly enhance patient management by offering dynamic insights into fluid status and overall cardiac function, but additional research in diverse settings is necessary to establish their clinical utility [15][16][17].

Moreover, the work of Olgin et al., [18] Just et al., [27] Golbus et al., [20] and Khandwalla et al. [21] emphasizes the critical need for improving wearable devices and their integration into comprehensive HF managment. While WCDs and activity sensors show promise in preventing sudden cardiac death and assessing treatment impacts [18], respectively, there are still gaps in design, data standardization, and patient adherence that must be addressed. Enhancing the accuracy of these devices and ensuring their seamless incorporation into existing treatment protocols could pave the way for more personalized and effective HF management strategies, ultimately leading to better patient outcomes [18][20][21][27] However, to fully realize the potential of these devices, it is essential to address the existing challenges and research gaps through collaborative efforts between clinicians, researchers, policymakers, and technology developers [26]. By doing so, we can ensure that wearable technology becomes an integral and effective component of HF management, ultimately improving the quality of life for patients living with this chronic condition [11].

Future research must focus on addressing these challenges to fully harness the benefits of wearable technology in HF management, ensuring it can be effectively and reliably incorporated into clinical protocols to improve patient outcomes.

## Conflict of Interest

None.
